# Virtual patient simulation in an interactive educational module on intimate partner violence: nursing students’ experiences—a mixed-methods study

**DOI:** 10.3389/fdgth.2025.1516379

**Published:** 2025-04-11

**Authors:** Joachim Eckerström, Sophie Mårtensson, Margaretha Larsson, Rajna Knez, Madeleine Ljudvåg, Samir El Alaoui, Karin Dahlström, Ylva Elvin Nowak, Terese Stenfors, Nitya Jayaram-Lindström, Marianne Kristiansson, Uno Fors, Karolina Sörman

**Affiliations:** ^1^ Centre for Psychiatry Research, Department of Clinical Neuroscience, Karolinska Institutet & Stockholm Health Care Services, Stockholm, Sweden; ^2^ Division of Nursing, Department of Neurobiology, Care Sciences and Society, Karolinska Institutet, Huddinge, Sweden; ^3^ Institution of Health Sciences, University of Skövde, Skövde, Sweden; ^4^ Department of Psychiatry, Skaraborg Hospital, Skövde, Sweden; ^5^ Institute of Neuroscience and Physiology, Sahlgrenska Academy, University of Gothenburg, Gothenburg, Sweden; ^6^ Academic Primary Health Care Centre, Region Stockholm, Stockholm, Sweden; ^7^ Department of Women's and Children's Health, Karolinska Institutet, Stockholm, Sweden; ^8^ Division of Family Medicine and Primary Care, Department of Neurobiology, Care Sciences and Society, Karolinska Institutet, Huddinge, Sweden; ^9^ Department of Learning, Informatics, Management and Ethics, Karolinska Institutet, Stockholm, Sweden; ^10^ Department of Computer and Systems Sciences (DSV), Stockholm University, Stockholm, Sweden

**Keywords:** virtual patient simulation, digital health education, digitalization, intimate partner violence, nursing students, mental health, simulation-based learning, online learning

## Abstract

**Background:**

Multiple studies have shown that healthcare professionals often feel uncertain about when to inquire about intimate partner violence (IPV), the appropriate methods for doing so, and how to respond to the answers. Virtual patient (VP) cases are an interactive educational tool that can be effective for learning and training clinical reasoning skills. However, there is a lack of research on the use of VP in psychiatry education. This study aimed to investigate nursing students' experiences of using a VP as part of an educational module, integrated into their foundational training on IPV during their nursing education.

**Methods:**

The study employed a mixed-methods approach, incorporating both quantitative and qualitative data. Participants (*N* = 62) completed an interactive educational module on IPV, in three consecutive parts: (a) a web-based education on IPV, (b) training with a VP, and (c) a seminar for follow-up discussions.

**Results:**

The VP platform was considered user-friendly and easy to navigate, although some participants found the instructions challenging. Participants perceived the VP as beneficial for learning about IPV and for practicing interactive patient dialogues. They appreciated the rich set of questions and the feedback provided, both by the experts in the field of IVP and by the VP itself. However, some participants noted that interacting with a VP on a screen was less emotional compared to real human interactions.

**Conclusion:**

The interactive educational module, integrated into the regular nursing program, was positively received by the students. Overall, the VP was considered beneficial for learning about IPV, easy to navigate, and provided a valuable opportunity for practice.

## 1 Introduction

Virtual patients (VP) are interactive, screen-based simulations of real-life clinical scenarios, predominantly used for training clinical reasoning and assessment (1). In nursing educations, simulation based learning as a learning strategy is increasingly used (2). In a recent scooping review on different methods of simulation based learning to foster critical thinking among nursing students, one key finding was that realistic features in simulated patients are beneficial for students learning and critical thinking. This led authors to recommend that realism should be a primary strategy when designing simulation based tools, taking into consideration both psychological, physiological and conceptual factors (2). VPs typically consist of an interactive platform where users digitally “meet” credible patient cases and practice their clinical interviewing and decision-making skills. Although VP designs vary, a common format provides users with clinically relevant background, allowing them to perform an assessment of the virtual patient, including medical history taking and receiving feedback on the actions taken. One advantage of VP as a tool is its ability to convey the complexity of a real patient through behavioral and facial expressions, as well as emotional cues (3). Such nuances can be difficult to teach through more traditional methods such as classroom-based instruction where vignettes are commonly discussed. VP has been used as an educational tool in healthcare for continued education of clinicians for many years (4), with research on its use accumulating since the 1990s. While it has been explored in various medical fields, knowledge is still limited regarding the use of VP in psychiatric education (5). In Sweden, VP has successfully been used as a training platform for clinical questions concerning refugees with trauma (6). International research indicates that VP positively impacts learning and clinical reasoning, also influencing behavioral change through engagement and motivation (7, 8). A meta-analysis showed that VP can be more effective than traditional education in enhancing clinical decision-making and critical thinking (4). In nursing education specifically, VPs are increasingly used as one form of simulation method, to enhance knowledge, clinical reasoning and decision-skills (9, 10). A systematic review on randomized controlled trials of VPs in nursing educations involving 787 students in total from different settgings, demonstrated that although design features of VPs and study qualities differed, overall VPs were efficient in enhancing student self-efficacy, knowledge, and nursing competencies (11). Studies on the use of VP representing different clinical areas, have shown effectiveness in enhancing nursing students' readiness for their clinical placements within psychiatry (12). Additionally, a recent review from medical and nursing programs reported various learning outcomes (e.g., communication skills, attitudes, stigma) in 5,563 students participating in undergraduate psychiatry education (5). Overall, these students perceived VPs as pedagogical models representing safe learning environments, with both the authenticity of the VP and integrative feedback being highly valued (5).

Given the proven effectiveness of VP in various educational contexts, there is significant potential to use this method to train healthcare professionals in recognizing and managing a wide range of clinical scenarios. One particularly suitable area for VP training is intimate partner violence (IPV), a pervasive issue that healthcare providers in many countries are responsible for identifying in order to offer appropriate care, support, and referrals (13, 14). IPV is a major public health concern and a violation of human rights, encompassing physical, psychological, and sexual violence (15). Globally, about one-third of women have been subjected to IPV at some point in their lives (15). In Sweden, a total of 4.7 percent of the population (ages 16–84) reported experiencing recurrent intimate partner violence in 2022, with 5.2 percent of women and 4.1 percent of men being repeatedly affected (16). Previous research indicates a correlation between exposure to violence and negative health consequences (17, 18). The acute injuries resulting from physical violence can be significant, but living with psychological violence and control has been shown in several studies to have serious consequences for both mental and physical health. For example, a study focusing on women with diabetes found that those who had been subjected to serious psychological violence in a close relationship had an 80 percent higher risk of developing type 2 diabetes than women who had not been exposed to violence (19). Unexplained somatic pain has also been shown in studies to have a very strong connection to psychological violence exposure in particular (20, 21). There is extensive research demonstrating strong correlations between exposure to IPV and various mental health conditions including depression, anxiety, post-traumatic stress disorder, suicidality, and substance abuse (15, 22, 23). Despite this, multiple studies have shown significant shortcomings in identifying victims of violence, and healthcare professionals often feel uncertain about when to ask questions, how to do so, and how to act on the responses (13, 14, 24). Barriers to posing questions about IPV can include cultural differences, lack of time, language barriers and importantly inadequate training (25). Nurses can be the first line of contact in health care settings, and they can have a vital role in identifying IPV and referring patients subjected to IPV. Therefore, it is vital that they are provided with adequate training with the latest knowledge on IPV and how it can be manifested in different patients. Adequate training on IPV is insufficient in nursing education programs, and the programs could be improved by strengthening nurses' communication skills and their ability to refer exposed patients (25).

Considering the difficulties in recognizing and asking questions about IPV in healthcare settings, efficient and tailored educational interventions are needed. A systematic review demonstrated that some key factors for effective programs included the inclusion of an online training component, delivered by an expert within IPV (14). Another review, which aimed to map out the limitations of curricular content in training healthcare professionals to address IPV, recommended strengthening the curriculum on IPV by allocating more time, improving the capability of teaching IPV, enhancing the course content, and establishing clear assessment methods (26). A meta-analysis of the effectiveness of digital education for healthcare professionals on domestic violence found low-quality evidence that such education can improve knowledge and self-efficacy, and recommended further research on digital interventions in this area (27). The educational interventions varied, including multiple formats such as theoretical lectures, seminars, interactive workshops, and simulated patient scenarios. Success factors for effective learning and understanding of how to integrate theoretical knowledge into practice included interactive formats and practical applications. The results also demonstrated that longer interventions, involving several in-depth sessions, were more effective than shorter ones (28).

In summary, previous research has indicated that VP can be a promising educational method for improving users' critical thinking, clinical skills, and decision-making. However, there is a lack of research on the use of VP in psychiatry training and education, including the field of nursing. Therefore, studies are needed to investigate the learning outcomes of VP among nursing students. Given the clear challenges healthcare staff face in asking questions about IPV, this topic could be a suitable area for practicing patient communication. The aim of the present study was to investigate nursing students' experiences of using a VP as part of their education on IPV. The following research questions were addressed: (1) How do nursing students perceive the user-friendliness of the VP? (2) How do nursing students experience the impact of the VP on their learning?

## 2 Materials and methods

### 2.1 Study design

This study employed a mixed-methods approach, incorporating both quantitative and qualitative data collection and analysis (29). This approach was executed through a convergent design, where quantitative and qualitative data were collected in parallel, analyzed separately, and then compared or combined to provide a more comprehensive understanding of the research problem.

### 2.2 Interactive educational module

Participants completed an interactive educational module on IPV, which consisted of three consecutive parts: (a) an interactive web-based education on IPV, (b) training with a VP, and (c) a seminar for follow-up discussions.

#### 2.2.1 Interactive web-based education

The web-based education on IPV for healthcare professionals was developed by the Academic Primary Health Care Centre in Region Stockholm, Sweden, with the aim of equipping healthcare professionals with the necessary knowledge and skills to identify and support patients exposed to IPV. Primarily targeting clinical staff, the education is also widely integrated into medical and nursing programs. The education, which takes approximately 2.5 h to complete, includes modules on the prevalence of IPV in the population and in healthcare settings, different types of abuse, psychological processes that perpetuate abusive relationships, health consequences of IPV, how to ask patients about IPV, the healthcare system's role in supporting victims of violence, and secure documentation practices in medical records. A variety of learning formats are employed, including factual texts, videos, and interactive elements such as multiple-choice questions (30).

At the University of Skövde, nursing students in semester 4 are expected to describe the implications of adverse health effects in relation to IPV as part of their course objectives. While this topic has not been explicitly examined in previous course plans, students may have encountered it during clinical placements or in literature related to health issues. Before engaging with the virtual patient (VP) module, students are required to complete the web-based IPV training, ensuring they have a foundational understanding of the subject. Additionally, they receive a short introduction to the VP module, including an overview of its purpose, structure, and intended learning outcomes. This introduction may consist of a briefing session, supplementary reading materials, or discussions to ensure students are adequately prepared before engaging with the virtual patient cases. The VP module is designed to enhance students' clinical reasoning and communication skills through realistic patient cases, where they practice identifying signs of IPV, asking appropriate questions, and planning suitable interventions.

#### 2.2.2 Training with a virtual patient

**Figure 1:**
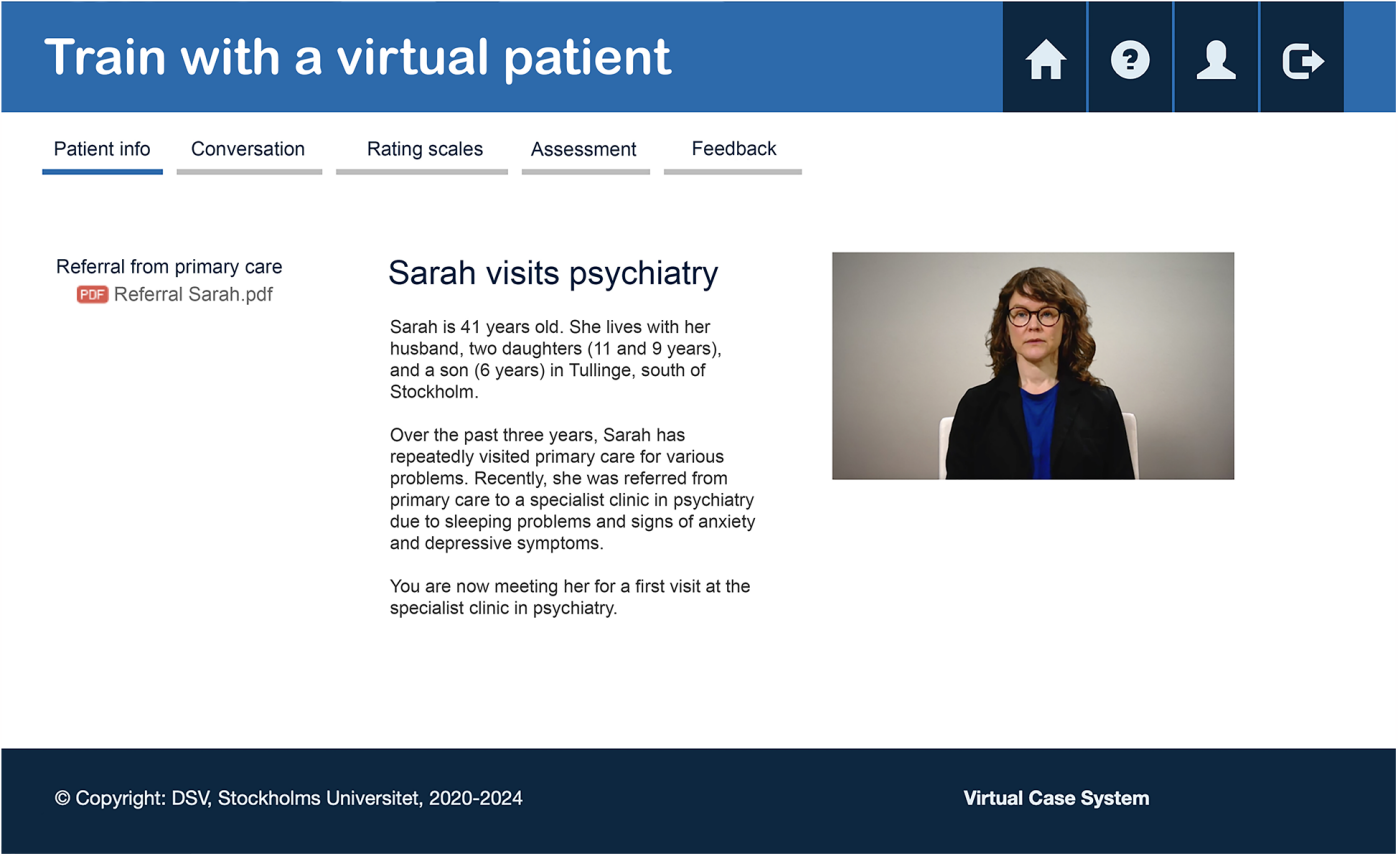
The platform for the virtual patient.

Based on Swedish and international research demonstrating positive learning effects and behavioral changes in users following training with VP, members of the current research group (JE, KD, YEN, MK, UF, KS) developed a VP specifically tailored for psychiatry, particularly focusing on IPV. This was the second VP on IPV that the group developed, with the previous one targeted towards primary care. The system used to develop and run the VP cases was the Virtual Case System (VCS), created at Stockholm University in Sweden. In VCS, a skilled teacher or clinical expert can easily create and edit VP cases without needing advanced programming skills. The dialogue section is based on video clips of a real actor to visualize body language, gaze, and tone of voice of a patient in distress. A professional actor was engaged to record the video clips. The VP is accessed through an online platform where users log in to a “virtual health center.” The VP used in the current study, illustrated in Figure 1, portrays a 41-year-old woman named Sarah, presenting with sleeping problems, anxiety, and depressive symptoms.

**Figure 2:**
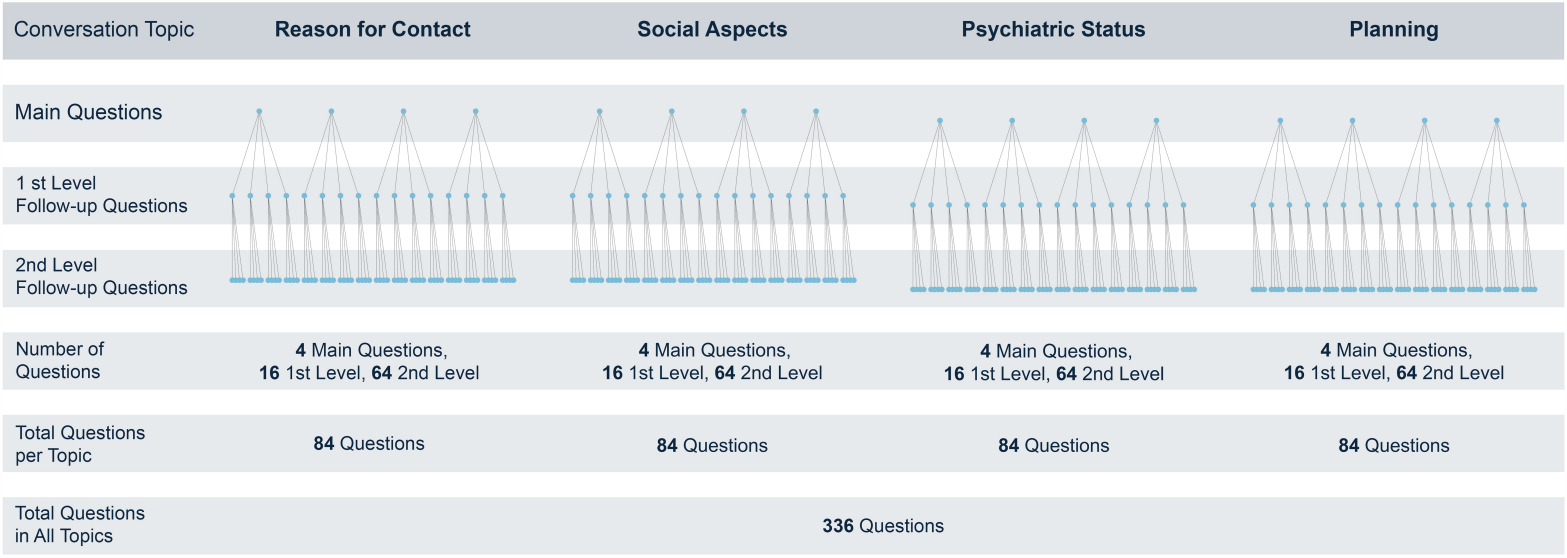
The structure of questions in the conversation with the virtual patient.

The virtual patient case is divided into several sections: (1) *Patient information*, where users are provided with information about why Sarah is referred to the psychiatric unit prior to the simulated consultation. (2) *Patient conversation*, which involves a dialogue with Sarah structured around four conversation topics: reason for contact, social aspects, psychiatric status, and planning. Within each topic, the user selects a main question from multiple options. The VP then responds through a pre-recorded video clip, and the dialogue progresses with subsequent follow-up questions in two levels. The patient case used in this study includes a base of approximately 336 questions. See Figure 2 regarding the structure of the questions. (3) *Rating scales*, the user can review the clinical rating scales that Sarah completed before the meeting, including Generalized Anxiety Disorder 7 (GAD-7) (31), Patient Health Questionnaire 9 (PHQ-9) (32), and Alcohol Use Disorders Identification Test (AUDIT) (33). (4) *Assessment*, where the user writes their assessment of the Sarah's current status, determines necessary actions, and identifies the need for follow-up. (5) *Feedback*, where the user receives automated feedback from both the VP and the experts who developed it, once everything is completed. This feedback is based on the user's choice of questions during the dialogue, with each question being graded from −1 (inappropriate) to +1 (appropriate). In the feedback from the VP, the experience is described based on the treatment received, trust, the structure of the conversation, and its overall value. The experts provide feedback based on categories relevant to the focus area, such as validation, open exploration, blame, information measures, and the appropriateness of questions posed within the specific context of IPV. A typical session with the VP takes between 30 and 60 min.

#### 2.2.3 Seminar for follow-up discussions

After completing the web-based education and VP training, students participated in a teacher-led seminar aimed at fostering reflective discussion and consolidating their learning. The seminar took place on-site, with students working in small groups of 6–12 participants. This setting provided an opportunity for them to share and discuss questions or challenges that emerged from their individual learning experiences. An experienced instructor guided these discussions, providing expert feedback and facilitating peer learning. This collaborative format helped bridge the gap between theoretical instruction and practical application in managing IPV cases. Examples of questions raised by students during the seminar included: “How can one work with the perpetrator of the violence?”, “How do you talk to and support the victim if they express love for the perpetrator?”, and “How would you attempt to redirect the conversation if you felt the patient was starting to question you and your questions?”. The seminar lasted approximately 1.5 h. The pedagogical foundation of this simulation-based training approach was grounded in Experiential Learning Theory (34).

### 2.3 Data collection

The survey was developed to capture both quantitative and qualitative insights (29). The design enabled the collection of standardized, comparable measures (e.g., user-friendliness, clarity of instructions, overall VP experience) alongside open-ended reflections on the educational module's strengths and areas for improvement. Data collection took place in January, 2024 at the end of the seminar.

#### 2.3.1 Sampling strategy & recruitment

Fourth-term undergraduate nursing students were selected for this study because their course objectives include key learning outcomes related to IPV and communication. To minimize potential influence from the research team, students were recruited by an independent teacher who had no active role in the course or study design. Participation was entirely voluntary, and students were informed that their decision to participate or decline would have no impact on their coursework or assessment. To further reduce the potential for coercion, participation was anonymous, and students completed the survey independently.

#### 2.3.2 Qualitative measures

To explore deeper perceptions and experiences, open-ended questions prompted participants to describe the advantages and disadvantages of training with a VP, as well as reflect on applying these skills in clinical practice. These responses provided context for the quantitative ratings by offering insights into why certain platform features were considered helpful and how the VP format might be improved. Prior to its administration, the survey was pilot-tested within the research team to ensure clarity and relevance of the items. Data collection and management were conducted via Research Electronic Data Capture (REDCap), hosted at Karolinska Institutet. The study received ethical approval from the Swedish Ethical Review Authority (#2023-06687-01). Participants provided written, informed consent before completing the survey, and all data were collected through the secure REDCap platform. To protect confidentiality, open-ended responses were anonymized, with unique identifiers and potentially sensitive details removed before analysis.

#### 2.3.3 Quantitative measures

For the quantitative component of the survey, Likert-scale and slider-scale questions were employed to assess various dimensions of the VP educational method. Participants rated their ability to navigate the VP platform on a scale from 1 (difficult) to 10 (easy), evaluated the clarity of instructions on a scale from 1 (difficult to understand) to 10 (easy to understand), and provided ratings for the logic of feedback from both the VP and experts on a scale from 1 (unclear) to 10 (clear). Additionally, they offered an overall rating of the VP as an educational method on a scale from 1 (poor) to 10 (very good) and indicated how well the web-based IPV module, VP, and teacher-led seminar complemented one another.

### 2.4 Participants

Participants were nursing students from the University of Skövde enrolled in their fourth semester. The nursing program spans six semesters in total, culminating in a bachelor's degree.

### 2.5 Data analysis

#### 2.5.1 Qualitative analysis

A qualitative content analysis, following the method outlined by Graneheim and Lundman (35), was performed on participants'open-ended responses. The total text analyzed consisted of 47 pages of responses. This method systematically categorizes and interprets textual data into similarities and differences by identifying units of meaning, creating codes, and grouping these codes into subcategories and categories that align with the study's objectives. Four researchers (SM, ML, MLj, RK) read the responses multiple times to ensure immersion, then collaboratively coded the transcripts, discussing discrepancies until reaching consensus. This approach provided a structured framework for capturing both the explicit and latent content of participants' experiences. In the next step, meaning units related to the aim of the study were identified and further reduced to simplified statements labeled with a code. Codes with similar meanings, or which dealt with the same topics, were grouped into subcategories, each of which was given a descriptive name. The subcategories with similar meanings were then grouped into three categories. Finally, the three categories were named with content characteristic words. To enhance trustworthiness (36), all involved authors (SM, ML, MLj, RK) participated in the entire analysis process and discussed the findings together. The analysis followed an iterative process, moving back and forth between the total text and parts of the text.

#### 2.5.2 Quantitative analysis

For the quantitative analysis, JASP software (version 0.18.3; 43) was used to generate descriptive statistics, including means, standard deviations, frequencies, and percentages, as well as to calculate Pearson's correlation coefficients. Variables were selected based on research questions related to user-friendliness and the perceived impact of the virtual patient (VP), including: (1) years of clinical experience; (2) self-rated digital competence; (3) perceived ease of navigating the VP platform; (4) perceived training value; (5) overall VP experience grade; (6) clarity of platform instructions; (7) perceived logic of patient feedback; (8) perceived logic of expert feedback; and (9) interest in using VPs again. The primary focus was on understanding how these variables relate to one another, as user interface, feedback mechanisms, and prior experience could each influence participants' attitudes and learning outcomes.

While this study was not anchored in a specific pedagogical framework, it aimed to explore these associations in an exploratory manner. Future research could benefit from incorporating a theoretical framework to further contextualize these findings. Pearson's correlation coefficients were computed, along with 95% confidence intervals (CIs) and Fisher's z effect sizes (with their standard errors) for each pair of variables. Correlations were interpreted using conventional benchmarks (e.g., |r| ≈ .10 as small, .30 as moderate, .50 as large).

## 3 Results

Out of a total of 76 students enrolled in the course, 62 participated, see Table 1. The results are organized based on the two research questions. The first section addresses user-friendliness, while the second section explores the educative learning experience.

**Table 1:** Participant background data.

Characteristics	*n* (%)/Value
Gender	*n* (%)
Male	5 (8.1)
Female	56 (90.3)
Prefer not to answer	1 (1.6)
**Total**	62 (100)
Age (years)	
Range	20–42
Mean	27.4
Standard Deviation	6.3
Median	26
Clinical experience of working within healthcare	*n* (%)
Yes	38 (61.3)
No	24 (38.7)
**Total**	62 (100)
Which field of work	*n* (%)
Psychiatric care	3 (7.9)
Somatic care	26 (68.4)
Other (e.g., elderly care)	9 (23.7)
**Total**	38 (100)
Number of working years in healthcare	
Range	1–15
Mean	5.3
Standard Deviation	3.4
Median	5
Experience asking about domestic violence	*n* (%)
Yes	7 (18.4)
No	31 (81.6)
**Total**	38 (100)
Experience of healthcare situations where this question was relevant but not asked	*n* (%)
Yes	11 (17.7)
No	51 (82.3)
**Total**	62 (100)
Digital skill level (self-rated on a 10-point scale, 1 = low, 10 = high)	
Range	1–10
Mean	6.5
Standard Deviation	2.2
Median	7
**Total**	60
Prior experience of virtual patients before this study	*n* (%)
Yes	5 (8.1)
No	56 (90.3)
Missing answer	1 (1.6)
**Total**	62 (100)

### 3.1 User-friendliness

The qualitative data provides insights into participants' perceptions and experiences concerning the user-friendliness of the VP platform. The analysis elucidates both the positive aspects, and the initial challenges encountered by the users.

#### 3.1.1 Easy to navigate

The qualitative analysis of user-friendliness revealed a predominantly positive perception of the VP platform. Participants expressed appreciation for the clear instructions and the ease of navigating through different sections to engage in dialogue with the VP. The branched questions within the dialogue were generally perceived as concrete and varied, contributing to a better understanding of the platform's structure. As one student expressed:

“The clear instructions and the number of dialogue questions made it easy for me to understand the structure and helped me to know when to move forward in the conversation with the VP patient”.

#### 3.1.2 Initial navigation challenges

Some participants highlighted challenges related to the clarity of instructions, particularly at the initial stages of engagement with the platform. These participants found it difficult to discern when a step in the dialogue was completed and when to advance to the next step. They also noted confusion regarding the visibility of unselected questions and whether they could revisit them. One student described the challenge:

“It was difficult for me to know when a step was finished, and it was time to move on to the next step. I thought the structure of the dialogue questions was unclear as the question I didn’t choose didn’t disappear, at the same time I wasn’t sure if I was allowed to go back and ask the questions I didn’t choose”.

Despite these challenges, participants’ interest in exploring all tabs and functionalities within the VP platform was evident, reflecting their engagement with the platform's features.

Quantitative data supported these qualitative findings, with participants generally rating their experiences positively across various aspects of user-friendliness (see Table 2).

**Table 2:** Participant experiences regarding user-friendliness.

User-friendliness						
Questions Self-rated on a 10-point scale	Total (*n*)	Missing (*n*)	Range	Mean	SD	Median
How did you find **navigating** the VP platform? (1 = difficult, 10 = easy)	59	3	1–10	6.7	2.2	7
How did you find the **instructions** on the VP platform? (1 = difficult to understand, 10 = easy to understand)	59	3	3–10	7.1	2.2	8
How did you find the logic in the **feedback from the patient**? (1 = unclear, 10 = clear)	59	3	2–10	7.3	2.1	8
How did you find the logic in the **feedback from the expert**? (1 = unclear, 10 = clear)	59	3	2–10	7.8	2.0	8
How would you rate your **experience** of using virtual patients? (1 = poor, 10 = very good)	59	3	5–10	8.2	1.6	8

### 3.2 Educative learning experience

The qualitative data provides insights into participants' experiences with the VP platform, highlighting both its educational value and the challenges encountered during practice.

#### 3.2.1 Valued practice opportunity

Qualitative analysis revealed that the majority of participants viewed the dialogue questions within the VP platform as highly educative. They appreciated the opportunity to practice conversing about the sensitive topic of IPV in a safe environment without risking harm to real patients. As one student reflected:

“The dialogue questions in the VP platform gave me an opportunity to practice how to manage and care for the VP patient. I felt safe asking these questions as she was not a real person”.

Participants noted that the slight variations in the dialogue questions provided valuable insights into how questions could be asked and how different formulations elicited diverse verbal and non-verbal responses from the VP patient. As one student expressed:

“When I asked the “wrong” dialogue questions to the VP patient, I immediately saw that the expression became more passive. This was great”.

Another student reflected:

“I felt that the VP became almost offended when the dialogue question was asked in one way, but by reformulating the dialogue question, the expressions became slightly different”.

This experiential learning was considered valuable for developing skills in patient management and care. However, a minority of participants expressed a desire for the ability to create their own dialogue questions, suggesting a potential area for improvement in the VP platform. As one student stated:

“If I had been allowed to ask my own questions and received written feedback on them, I think I probably would have learned more”.

After completing all steps in the VP platform, the students received written feedback from the VP and from experts in IPV. Most participants stated that they valued the written feedback from the experts more than the feedback from the VP. Participants expressed that the written feedback deepened their awareness of their own strengths and limitations and thus helped them to reflect on their development in becoming a professional nurse. As one student described:

“After the written feedback, I redid the dialogue with the VP patient to see if I carried out the conversation differently, and yes, I did”.

The undergraduate nursing students expressed that practicing with the VP platform added a novel dimension to their learning experiences on IPV, far beyond what traditional lectures and course literature can provide. In the VP platform, they had the opportunity to actively engage with the VP's verbal and non-verbal expressions. In contrast, a minority of participants found practicing with the VP platform sometimes challenging, as they were not able to convey compassion using their own verbal or bodily expressions to comfort the VP. As one student reflected:

“The dialogue with the VP patient felt a bit emotionless because it took place through a computer screen”.

Quantitative data supported these findings, with participants generally rating their experiences positively across various aspects of the educative learning process (see Table 3).

**Table 3:** Participant experiences regarding the integrated educational program as educational learning modules.

Educative learning experiences						
Questions Self-rated on a 10-point scale	Total (*n*)	Missing (*n*)	Range	Mean	SD	Median
How relevant did you find the **digital web-based education** on intimate partner violence that you completed before using the VP? (1 = Not at all, 10 = very)	55	7	2–10	8.6	1.9	9
Do you find the **training with the virtual patient** beneficial for your learning about intimate partner violence? (1 = Not at all, 10 = very)	55	7	2–10	8.4	2.0	9
How valuable was the **dialogue-based seminar** for your learning about intimate partner violence? (1 = Not at all, 10 = very)	55	7	5–10	8.8	1.3	9
How well do you think the different **learning components** (digital web-based education, virtual patient, and dialogue-based seminar) were integrated and complemented each other? (1 = Not at all, 10 = very)	55	7	5–10	8.7	1.4	9
How interested are you in **using virtual patients again** as an educational method (regarding another subject)? (1 = Not at all, 10 = very)	59	3	5–10	8.5	1.6	9

**Figure 3:**
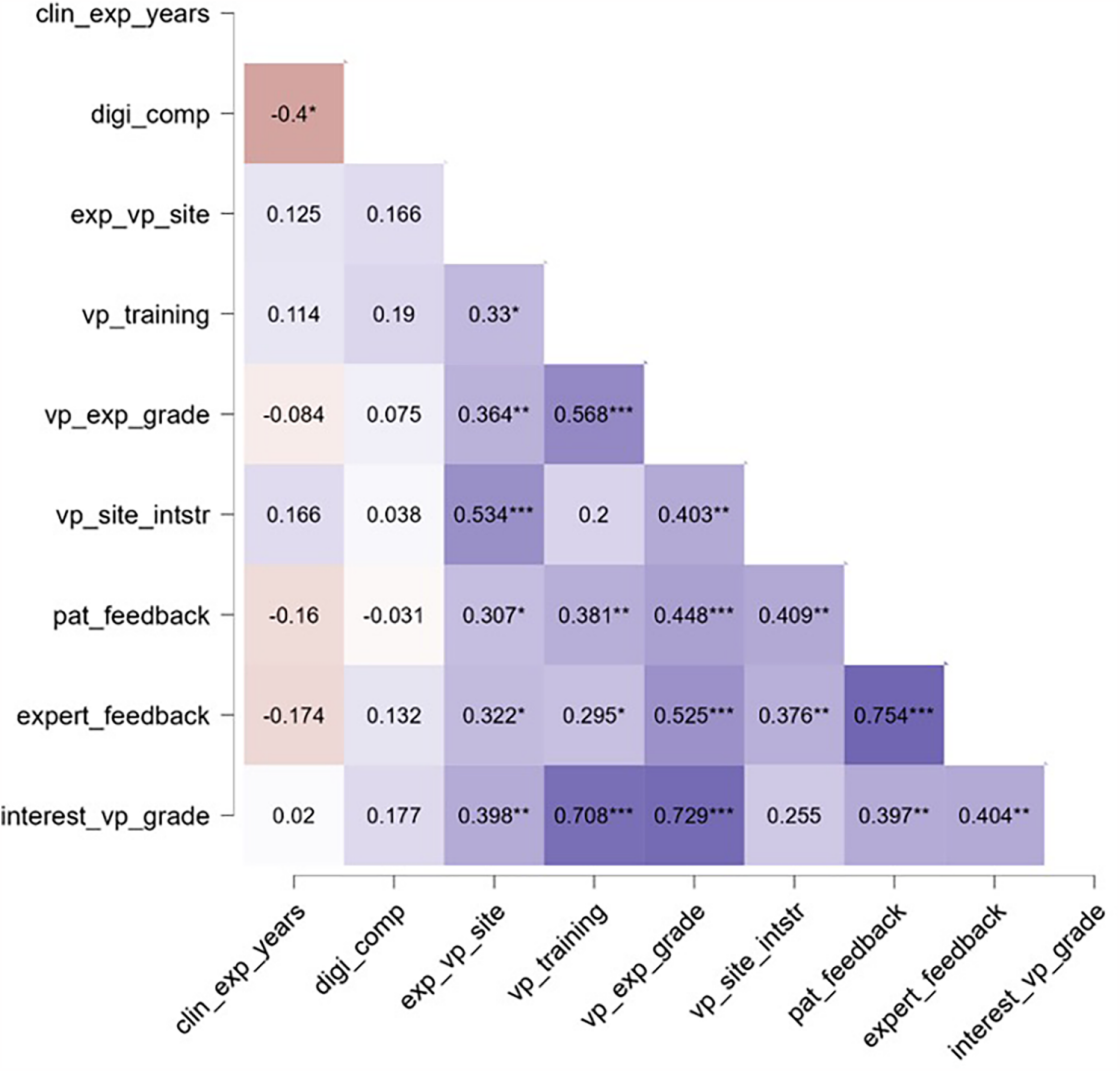
Correlation matrix of key study variables. Note. *p < .05, **p < .01, ***p < .001. clin_exp_years, years of clinical experience; digi_comp, self-rated digital competence; exp_vp_site, perceived ease of navigating the VP platform; vp_training, training value; vp_exp_grade, VP experience grade; vp_site_intstr, clarity of platform instructions; pat_feedback, perceived logic of patient feedback; expert_feedback, perceived logic of expert feedback; interest_vp_grade, interest in using VPs again.

The correlation analysis (Table 4) revealed multiple associations among the key study variables (Figure 3). Years of clinical experience correlated negatively with self-rated digital competence [*r* = −0.40, *p* < .05, 95% CI (−0.64, −0.08), Fisher's z = −0.42, SE = 0.17]. Perceived ease of navigating the VP platform (“exp_vp_site”) showed positive relationships with training value [*r* = 0.26, *p* = .05, 95% CI (−0.00, 0.48), z = 0.26, SE = 0.14], overall VP experience grade [*r* = 0.36, *p* < .01, 95% CI (0.12, 0.57), z = 0.38, SE = 0.14], clarity of platform instructions [*r* = 0.53, *p* < .001, 95% CI (0.32, 0.70), z = 0.60, SE = 0.14], perceived logic of patient feedback [*r* = 0.31, *p* < .05, 95% CI (0.05, 0.52), z = 0.32, SE = 0.14], and perceived logic of expert feedback [*r* = 0.32, *p* < .05, 95% CI (0.07, 0.54), z = 0.33, SE = 0.14]. Collectively, this pattern suggests that a user-friendly interface is associated with more positive evaluations of the learning experience.

**Table 4:** Correlation matrix of key study variables.

Variable	Statistic	clin_exp_years	digi_comp	exp_vp_site	vp_training	vp_exp_grade	vp_site_intstr	pat_feedback	expert_feedback
digi_comp	r	−0.400*	—						
	z	−0.424	—						
exp_vp_site	r	−0.138	−0.159	—					
	z	−0.139	−0.160	—					
vp_training	r	0.409**	0.376**	0.255	—				
	z	0.434	0.396	0.261	—				
vp_exp_grade	r	−0.084	0.075	0.364**	0.568***	—			
	z	−0.084	0.076	0.381	0.644	—			
vp_site_intstr	r	0.166	0.038	0.534***	0.200	0.403**	—		
	z	0.167	0.038	0.596	0.203	0.427	—		
pat_feedback	r	−0.160	−0.031	0.307*	0.381**	0.448***	0.409**	—	
	z	−0.161	−0.031	0.317	0.401	0.483	0.434	—	
expert_feedback	r	−0.174	0.132	0.322*	0.295*	0.525***	0.376**	0.754***	—
	z	−0.175	0.133	0.334	0.304	0.583	0.396	0.981	—
interest_vp_grade	r	0.020	0.177	0.398**	0.708***	0.729***	0.255	0.397**	0.404**
	z	0.020	0.179	0.421	0.883	0.926	0.261	0.420	0.428

For each correlation, *r* = Pearson's correlation coefficient; *z* = effect size (Fisher's z). clin_exp_years, years of clinical experience; digi_comp, self-rated digital competence; exp_vp_site, perceived ease of navigating the VP platform; vp_training, training value; vp_exp_grade, VP experience grade; vp_site_intstr, clarity of platform instructions; pat_feedback, perceived logic of patient feedback; expert_feedback, perceived logic of expert feedback; interest_vp_grade, interest in using VPs again.

* *p* < .05.

** *p* < .01.

*** *p* < .001.

In addition, training value (“vp_training”) exhibited strong correlations with overall VP experience grade [*r* = 0.57, *p* < .001, 95% CI (0.35, 0.73), z = 0.64, SE = 0.14] and with interest in using VPs again [*r* = 0.71, *p* < .001, 95% CI (0.54, 0.82), z = 0.88, SE = 0.14]. Likewise, overall VP experience correlated strongly with interest in future VP use [*r* = 0.73, *p* < .001, 95% CI (0.58, 0.83), z = 0.93, SE = 0.14]. These findings suggest that participants who perceived the VP training as particularly valuable also tended to rate their overall experience more favorably and expressed a heightened interest in using VPs again. Finally, perceived logic of patient feedback correlated strongly with perceived logic of expert feedback [*r* = 0.75, *p* < .001, 95% CI (0.62, 0.85), z = 0.98, SE = 0.14], highlighting the interconnected nature of these two feedback components.

## 4 Discussion

The present study investigated nursing students' experiences of using a VP as part of an interactive educational module on IPV. The results provide insights into key factors that could influence the effectiveness of integrating VPs into healthcare educational programs. A primary finding of this study is that the VP was considered beneficial for learning about IPV. The generally positive self-ratings of the three-part educational module (i.e., a web-based education on IPV, training with the VP, and a seminar for follow-up discussions) indicate that the multifaceted blended-learning model was well-received. Triangulating different learning modalities leverages their respective strengths and reinforces the acquisition of both theoretical and practical skills (37).

Another main finding was the overall positive perceptions of user friendliness of the platform. The design of VPs varies widely, and especially the possibility of an interactive illness history taking with or without video-based answers (38). Our VP platform includes a conversation section with several levels of questions and follow-up questions, and an elaborated feedback structure (i.e., with feedback from both the VP and an expert, based on the choice of questions in the conversation). The branched questions seemed to have provided the nuances that are valuable to users. This is probably one of the key features where VPs can be an important add on to more theoretical lectures—nuances in clinical situations are probably difficult to convey in traditional classroom-based teaching.

Perceptions of the interaction with the VP provided valuable insights into its design features. While several students observed emotional and facial cues of the VP in response to their questions, others described the experience as “emotionless”, indicating a lack of emotional expressiveness that may diminish both realism and learner engagement. Recent advances in AI-driven adaptive responses have demonstrated that technology can detect and respond to user affect—such as facial expressions, tone of voice, or body language—in real time (39). Incorporating these techniques could enable the VP to display more nuanced emotional reactivity, making it appear more lifelike and empathetic. Improved emotion modeling, for instance by integrating natural language processing with dynamic facial animation, could further encourage deeper immersion by mirroring the subtleties of human interaction. Future iterations of our VP will explore these avenues to better align with the emotional tone of real-life clinical encounters, ultimately enhancing the authenticity and pedagogical impact of VP simulations. This approach aligns with a recent systematic review on the use of VPs by medical students, which found that while students appreciated VPs for enhancing clinical reasoning, they also noted that these simulations lacked the complexity and interactivity of real patient encounters (40). Future developments may explore whether VPs developed with generative AI using Large Language Models (LLMs) to simulate a variety of psychiatric and somatic symptoms are perceived as more realistic, potentially enhancing both engagement and the overall learning experience (39).

The analysis revealed several noteworthy correlations. There was a positive association between the perceived usability of the VP platform (ease of navigation and clarity of instructions) and participants' overall ratings of the training value, feedback logic, and general VP experience. This aligns with previous research highlighting the importance of user-friendly interfaces and clear guidance for the successful implementation of digital learning tools like VPs (41). Poorly designed platforms could likely lead to frustration and disengagement from the educational content. Therefore, investing in well-designed, intuitive VP interfaces appears critical for maximizing potential learning benefits.

The strong positive correlations between perceived training value, overall VP experience, and interest in using VPs again point to the central role of the specific VP training case itself. When students find the training case valuable and engaging, it likely enhances their motivation, active participation, and ability to effectively apply their knowledge, all of which could boost the educational impact (42). Designing high-quality, relevant VP cases that integrate expertly crafted feedback seems key for fostering positive learning experiences that can transfer to clinical practice.

The negative correlation between clinical experience and self-rated digital skills suggests more senior clinicians may face barriers in digital competencies. This highlights the importance of user-centered implementation strategies and available technical support when introducing digital educational interventions. Some learner groups, particularly those with less digital experience, may benefit from additional, targeted training sessions focused on navigating the platform and building digital confidence to ensure they can fully engage with the educational content.

### 4.1 Developmental areas

One developmental area would be to expand the current platform to include several different types of VPs addressing varying backgrounds (i.e., age groups, gender, ethnicity, and cultural backgrounds). This would provide the user with a richer insight into how exposure to IPV can be manifested in patients depending on varying background factors. It would also be utilized to demonstrate how exposure to IPV can correlate with multiple health problems (including both somatic and psychiatric) in different individuals. In addition to several VP patients, the platform could also be expanded with (a) paediatric cases portraying children exposed to family environments with ongoing IPV and (b) perpetrators of IPV, and (c) different stakeholders who usually can get involved around cases of IPV (e.g., social services, police, correctional services). With such a rich plethora of training arenas, the educational module could work more as a scenario-based platform, where the user can gain knowledge and practice different pathways, around perpetrators and victims of IPV.

### 4.2 Limitations

The present study has several limitations. Participants were drawn from a single university program, limiting the generalizability of our findings. Future research would benefit from multi-institutional designs involving nursing students from diverse geographical and educational contexts. Such collaborative studies could offer stronger evidence regarding the effectiveness and adaptability of the VP module, ultimately supporting broader implementation within nursing curricula. Furthermore, the vast majority of participants were female, which made it challenging to identify any gender differences. Future studies should include multi-center settings with students from different programs and universities. Although an internal review of the survey was conducted to ensure clarity and content relevance, the instrument was not formally validated prior to this study—such as through cognitive interviews with nursing students or calculating reliability measures (e.g., Cronbach's Alpha). This constitutes a limitation of the study, and it is recommended that future research include a rigorous survey validation process before large-scale administration. Also, while the study employed a mixed-methods approach, incorporating both quantitative (self-reported) and qualitative data, this provides valuable insights into students' perceptions and subjective learning experiences, we acknowledge that reliance on self-reported measures can introduce social desirability bias. To more objectively gauge skill development and knowledge retention, future studies should consider employing validated pre- and post-tests or structured clinical assessments. Such methods would allow researchers to compare students' baseline proficiency with their post-training performance, thus providing a more robust measure of the VP module's effectiveness. The correlation analysis revealed multiple associations among variables related to user-friendliness and perceived training value. However, it is important to note that correlation does not imply causation, and these findings should be interpreted with caution. While students' perceptions of ease of navigation and expert feedback correlated with higher ratings of overall VP experience and interest in using VPs again, we cannot determine whether these factors directly cause improvements in learning or skill development. Future studies should employ longitudinal or experimental (e.g., randomized controlled trial) designs to establish causality and to more definitively determine whether the VP experience leads to enhanced competencies over time. Conducting cognitive interviews, where individual participants from the target group are asked to reflect on how they understand and interpret the survey questions, could have strengthened its use in the current study.

## 5 Conclusion

In conclusion, the study provides novel insights into key factors that may influence nursing students' experiences with VP-based education about the crucial topic of IPV. A user-centered approach focusing on platform usability, engaging and realistic training cases, expert-guided feedback, and blending different learning activities emerges as a promising educational model. Addressing potential digital skills gaps through targeted implementation strategies is also highlighted. Overall, the findings can guide efforts to effectively integrate interactive VP simulations into health professional training curricula.

## Data Availability

The raw data supporting the conclusions of this article will be made available by the authors, without undue reservation.
